# Measurement of the translation and impact from a childhood obesity trial programme: rationale and protocol for a research impact assessment

**DOI:** 10.1186/s12961-017-0266-9

**Published:** 2017-12-19

**Authors:** Penny Reeves, Simon Deeming, Shanthi Ramanathan, John Wiggers, Luke Wolfenden, Andrew Searles

**Affiliations:** 1 Hunter Medical Research Institute (HMRI) Lot 1, Kookaburra Circuit, New Lambton Heights, NSW 2305 Australia; 2 School of Medicine and Public Health, The University of Newcastle, University Drive, Callaghan, NSW 2308 Australia; 3 Hunter New England Population Health, Wallsend, NSW 2287 Australia

**Keywords:** Research translation, Outcome research, Outcome measurement, Public health policy, Impact assessment, Evaluation

## Abstract

**Background:**

There is growing recognition amongst health and medical research funders and researchers that translation of research into policy and practice needs to increase and that more transparency is needed on how impacts are realised. Several approaches are advocated for achieving this, including co-production of research or academic-practitioner research. The Population Health Unit (PHU) within the Hunter New England Local Health District in regional Australia, as an early adopter of this model, has been working to increase the likelihood that its research is translated into community health benefits. With the New South Wales Ministry of Health, the PHU responded to the burden of child overweight and obesity by combining service delivery with research expertise. The ‘Good for Kids, Good for Life’ (Good for Kids) dissemination trial was developed and implemented in seven community settings in the Hunter region of Australia between 2006 and 2010. This study aims to undertake a retrospective impact assessment to measure the research translation and impact of Good for Kids.

**Methods:**

The method will be based upon the application of the Framework to Assess the Impact from Translational health research (FAIT), comprising three core elements, namely quantified metrics, economic assessment and a narrative of the process by which the research in question translates and generates impact.

**Discussion:**

Increasingly, funders are interested both in the outcomes resulting from investments in health research and in the expected return on their investments. FAIT was developed specifically for this purpose and its use is anticipated to provide transparency to the pathway to translation and potentially drive increased investment in translational research programmes such as Good for Kids.

## Background

An expected outcome of funding health research is that research findings will contribute to societal improvement, in particular patient or community health and wellbeing. However, despite these expectations, 50% or more of research does not optimally translate, especially with respect to end users, in practice and policy [1, 2]. A consequence of research translation and impact being lower than it could be implies that the returns on research investments are not being fully realised.

In direct response to the disparity between the creation of research outputs and the uptake of those outputs, a team of health economists and health and medical researchers from the Hunter Medical Research Institute developed the Framework to Assess the Impact from Translational health research (FAIT). FAIT was created using mixed methods, including (1) a scoping review of existing research impact frameworks and techniques to inform its development, (2) design of the prototype and (3) a feedback stage where iterations of the prototype were presented to selected researchers for discussion and refinement [3]. The final Framework comprises three core elements, namely quantified metrics, economic assessment and a narrative of the process by which the research in question translates and generates impact. FAIT, primarily designed to be applied prospectively, is based on the premise that planning, monitoring and providing feedback about activities and behaviours associated with research translation should lead to successful translation. FAIT is currently being employed to measure and value the impact from research being conducted through two Australian federally funded Centres for Research Excellence, one in stroke rehabilitation and one in Indigenous primary healthcare. FAIT has not previously been applied to research related to child overweight and obesity.

The ‘Good for Kids, Good for Life’ (Good for Kids) dissemination trial provides another opportunity to apply FAIT’s impact assessment method, albeit retrospectively, to a different area – childhood obesity – and provide transparency to the translation pathway for Good for Kids. Given the anticipated time lag between research and impact [4], FAIT’s application to Good for Kids will allow greater testing of FAIT’s impact assessment methods.

Good for Kids was a novel, multi-setting, primary prevention approach to reducing the prevalence of child overweight and obesity and improving children’s healthy eating, physical activity and small screen time behaviours [5]. The trial was developed in response to the increasing incidence and prevalence of child overweight and obesity observed in the early 1990s. A child obesity summit was convened in the state of New South Wales (NSW) in late 2002, which provided both the mandate and foundation for subsequent state-based child obesity prevention programmes [6]. At the time, Good for Kids represented Australia’s largest ever community-based child obesity prevention programme. The programme was implemented by the former Hunter New England Area Health Service, now Hunter New England Local Health District (HNELHD), in partnership with a broad range of government, non-government organisations and private organisations. Specifically, the trial involved the implementation of separate interventions in seven different community settings, namely children’s services (e.g. day care centres), primary schools, community sports clubs, primary healthcare services (General Practice), community service organisations (non-government organisations providing home visiting services for vulnerable families with young children), Hunter New England health services, and Aboriginal health services. The setting-specific interventions sought to facilitate the adoption by the organisations of practices that promote child healthy eating and physical activity, as well as the implementation of supportive organisational policies, systems and procedures. The programme ran formally between 2006 and 2010, after which the component interventions became part of the NSW Government Healthy Children Initiative.

Good for Kids was set up as a translational research programme. As both practitioners and implementers of health research, the Population Health Unit of the Hunter New England Local Health District (HNEPH), an early adopter of research co-production [7], is seeking to introduce greater transparency to the health, social and economic impacts realised through its research and to better understand the factors that will continue to optimise these impacts. The retrospective application of FAIT is expected to meet these aims. The HNEPH research-practice partnership is focused on conducting health research assessing intervention effectiveness in addressing chronic disease risk and also trialling strategies to improve the implementation of evidence-based health interventions [7]. To date, the impact of research conducted by HNEPH has been measured in terms of direct health benefit or by the extent to which interventions have been adopted into Local Health District, State or National policies. Measuring the broader economic and social impact using FAIT is a natural next step in the provision of information relevant to policy-makers and funders.

This paper describes the research protocol of a study, based upon the retrospective application of FAIT, to measure and give transparency to the translation and impact of Good for Kids.

The current study has two aims. First, through the application of FAIT, to use available evidence to assess the research impact of Good for Kids and, second, to provide transparency to the pathway to impact.

## Methods

### Study design, population and recruitment

The study will employ a mixed methods approach to research impact assessment, involving three components. Component 1 involves two literature reviews that will be conducted to uncover any other published impact assessments on similar research programmes and to inform the type of benefits that may result from the research as well as to identify potential values or sources of value associated with those benefits. Component 2 will be the retrospective construction of a modified programme logic model of the Good for Kids trial, the purpose of which will be to provide an explanation of the linkages from knowledge generation to utilisation of the outputs generated by the research. Finally, component 3 will be impact measurement and valuation. The results will be summarised and presented by way of a scorecard, including illustrative case studies describing the process by which Good for Kids translated and generated impact. Detail on each of these components is discussed below.

The setting for this study is the PHU within HNELHD in NSW, Australia. The district is geographically large (131,785 km^2^), with an estimated population of almost 1 million residing in metropolitan urban and suburban areas, regional centres, and rural and isolated remote communities.

#### Literature reviews

To address component 1, the two scoping literature reviews will be conducted following the Joanna Briggs Institute guideline for scoping reviews [8]. While still methodical in their approach, scoping reviews are typically broader in their focus with less restrictive inclusion criteria than systematic reviews [9]. These two reviews will be used to map the key concepts underpinning the measurement of impact in child obesity research. As outlined in the Joanna Briggs Institute guideline, a three-step search strategy will be used. Step 1 will involve an initial search of two relevant online databases. This will be followed by an analysis of the text words contained in the title and abstract of any retrieved papers and of the index terms used to describe the articles. A second search will then be undertaken using all identified keywords and index terms across all included databases. Finally, the reference list of all identified reports and articles will be hand-searched for additional studies. In these reviews, literature will be drawn from both economic (i.e. Econlit and JStore) and general health and medical academic databases (i.e. Medline, CINAHL, Cochrane Database of Systematic Reviews). The searches will also extend to Greylit, Google Scholar and Google to identify literature from government departments, international organisations and research funders. The searches will be limited to articles published in English, between 1990 and 2017. This timeframe is considered to be appropriate for two reasons. First, an increase in the prevalence of child obesity was first observed in the early 1990s [10] and, second, knowledge translation, a precursor to impact assessment, first gained prominence in the 1990s. Since these are scoping reviews, a number of limitations apply. First, no formal assessment of the quality of the studies will be undertaken, consistent with the method for a scoping review [8]. Second, our searches might have missed studies not included in the searched databases and not easily available on the internet.

The data from each review will be charted to record the key information relevant to each review. In line with recommended scoping review guidelines, the charting of results will be iterative [9, 11, 12]. The tabulated results will be accompanied by a narrative aligned to the review objective. The implications of the findings from each review for the application of FAIT to Good for Kids will also be discussed, particularly in regard to informing the domains of benefit and valuations for inclusion in the economic assessment.

#### Modified programme logic model

**Fig. 1:**
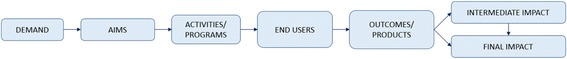
Modified programme logic model

The information garnered from the scoping reviews will inform component 2, a modified programme logic model [3] of Good for Kids (Fig. 1). The modification to the programme logic model relates to the inclusion of ‘end users’, which has the advantage for impact assessment purposes of identifying who will adopt the research outputs, including both interim and final users. The purpose of the model will be to retrospectively map the translation pathway for the interventions tailored to each of the seven community settings covered by the trial. The construction of the programme logic model will include (1) documenting the need that was being addressed by each of the Good for Kids interventions; (2) describing the research activities that were supplied to meet the need; (3) documenting the expected research outputs when the research was designed; (4) identifying the perceived end-users of those research outputs when the research was designed; and (5) describing the anticipated impacts from the use of the research outputs when the research was designed. The value in articulating these processes in the form of a retrospective programme logical model will be to give transparency to the research approach. Documents such as project plans, reports and meeting minutes will be used to inform the creation of the programme logic model, supplemented with semi-structured stakeholder interviews and feedback sessions with the main chief investigators (CIs). The modified programme logic map will form the basis upon which all aspects of the impact measurement will take place.

#### Impact measurement and valuation

Currently, there is no single measurement method capable of capturing the impacts stemming from health and medical research. For this reason, component 3 of FAIT employs a combination of three proven methods, namley quantified metrics [13], an economic assessment and a narrative of the process by which the research in question translates and generates impact.

##### Quantified metrics

Quantified metrics, based on the Payback Framework [13], will involve the identification of domains of benefit. A description and, where possible, measurement in natural units, of the relevant impacts for each domain will be made, elicited through the use of relevant supporting project documentation and semi-structured interviews with researchers [14]. In this retrospective application of FAIT to Good for Kids, these domains and examples of associated impacts will include advanced knowledge (e.g. number of PhD students associated with the research), clinical implementation (e.g. reduced availability of sweetened beverages in school and sporting club canteens, reduced availability of energy dense, nutrient poor foods in school and sporting club canteens, and greater allocation of physical activities in school and pre-school schedules), community benefit (e.g. reduced consumption of sweetened and non-sweetened drinks, reduced consumption of energy dense, nutrient poor foods, child consumption of vegetables and fruit, and child time spent in organised and non-organised physical activities), policy and legislation (e.g. policy spin-offs and sustained programmes), and economic benefit (e.g. reduced treatment costs associated with less chronic disease in the community). The quantified impact metrics will necessarily be limited to outcome measures selected at the research programme outset to measure the efficacy of the research components.

##### Economic assessment

The economic assessment component in this application of FAIT will entail a comparison of the costs associated with developing and implementing Good for Kids against a calculated value for the realised impact. The planned assessment will be modelled on both the Co-operative Research Council endorsed evaluation framework – the Impact Tool [15] and the Social Return on Investment Network Impact Map [16]. Both these approaches use cost benefit analysis as their foundation. Their appeal in guiding the economic assessment stems from their emphasis on the logic underpinning the research activity–output–usage–impact chain to give transparency and clarity, which is also at the heart of FAIT. The modified programme logic model is pivotal for articulating programme inputs, outputs, uptake and ultimate impact for each of the interventions. The calculated total costs and benefits will be combined by way of an impact map. Three broad steps will be involved in the economic assessment (1) Identification and measurement of resource use; (2) measurement and valuation of impacts and (3) comparison of the costs and benefits in a single metric. Where possible, the analysis will assume a societal perspective to ensure all possible costs and benefits are accounted for. The time horizon for the assessment will be bounded in the base case analysis by the period during which the programme received core funding, namely 2006–2010. Longer time horizons will be explored based on the time frames required to observe specific impacts. Costs and benefits will be reported in net present value terms and streams of projected future costs, and benefits will be discounted at a rate of 3% [17].

##### Identification, measurement and valuation of resource use

Guided by the modified programme logic model, resource use pertaining to (1) the development of the interventions, (2) delivery of the interventions, (3) uptake of outputs by end users, and (4) health outcome changes will be identified, measured and valued. The retrospective nature of this application of FAIT hampers the collection of data to inform some of the costs and benefits. This is especially the case for those costs incurred as a result of adopting or using the research outputs.

Resource use associated with development and delivery of the interventions will include the initial amount invested by both the NSW Ministry of Health and the local health district. Resource use costing will be based on the financial and administrative records provided by the CIs. The costs associated with the uptake of Good for Kids-related activities and programmes will include any costs (including opportunity costs) incurred by the various community organisations such as costs related to practice change. As stated above, it will be problematic to collect data to inform these costs retrospectively. However, some attempt will be made to model these costs using administration records and detailed descriptions of uptake obtained from CIs to inform the calculations. Unit costs for health service resource use will be as per the Medicare Benefits Schedule. Resource use of marketed goods and services outside the health sector will be valued at market prices. Unmarketed goods and services, such as travel time and the time of volunteer caregivers, will be valued using opportunity cost prices.

##### Measurement and valuation of impacts

As outlined above, based on the quantified metrics, those impacts that lend themselves to being valued in monetary terms will be included in the economic assessment. Examples of potential impacts specifically stemming from Good for Kids are increases in the proportion of children’s services with menus meeting dietary guidelines, adoption or scaling of the interventions outside the Hunter New England region, adoption of the interventions as routine practice by schools, and increases in physical activity levels in preschool and primary school aged children. The valuation of these impacts will involve projection and transformation to the economic benefits stemming from reduced prevalence of obesity and overweight in the community. Comorbidities linked to obesity and overweight, such as cardiovascular disease, some cancers and musculoskeletal disorders, are associated with significant demands on the healthcare sector in the form of direct costs associated with attendances to GPs, specialists, allied health, as well as hospital care [18]. Additionally, indirect costs are incurred in the form of productivity losses [19]. These economic costs are predominantly incurred by adult populations. The economic benefit directly attributable to Good for Kids will occur as a result of reducing the number of children who either remain or become obese or overweight into adulthood. All monetised benefits will be adjusted for attribution, the value of which will be informed by administrative and evaluation records and qualified during the CI interviews. Projected valuations will necessarily include a ‘drop-off’ factor to account for waning benefit over time.

##### Comparisons of costs and benefits in a single metric

Economic assessments, whether they are cost-effectiveness, cost-utility or cost-benefit analyses, commonly report results in terms of a single metric. For the purposes of this study, we will be reporting the results of the economic assessment in terms of a cost-benefit ratio. The total discounted benefits (research impacts) will be divided by the total discounted costs. A positive ratio (greater than 1) will indicate that the impacts outweigh the costs and the higher the ratio, the greater the impacts relative to the costs.

#### Narratives

The inclusion of illustrative examples or narratives will introduce a qualitative aspect to the measurement of research impact and provide an account of how translation occurred and how research impact was generated. Narratives or case-studies are part of the Payback Framework and are used as part of retrospective impact assessment [20]. In other applications of FAIT, these case-studies have been important vehicles for verifying the consistency of the impact findings generated from the economic assessment and the quantified metrics. In this application, it is expected that the examples will be informed by the programme logic and developed during the CI interviews.

#### Results reporting

##### Scorecard summary

**Fig. 2:**
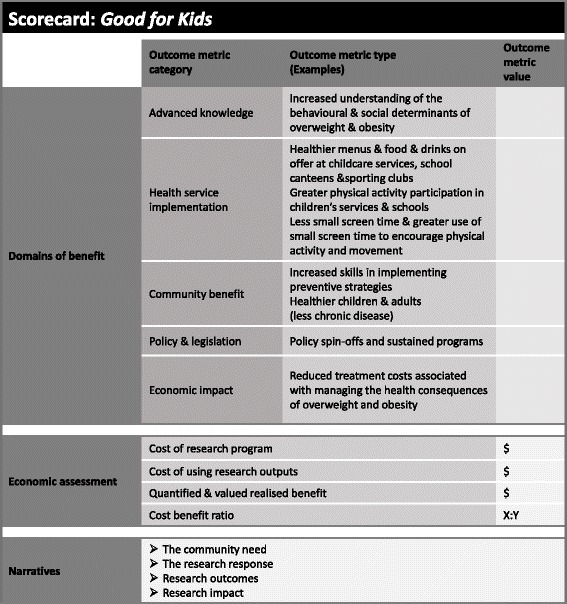
Proposed scorecard template

The results, including the narratives, will be summarised and reported by way of a scorecard. Figure 2 below presents the proposed scorecard template for the Good for Kids trial programme.

## Discussion

The dual aims of this study are to assess the research impact of Good for Kids using available evidence and to provide transparency to the pathway to impact. A key strength of the study is the application of a comprehensive impact assessment framework that incorporates three validated impact measurement methods. The timing of the assessment is also a strength given the expected time lag between research activity and the anticipated impacts, should the research translate. However, the time lag can also be a limitation, introducing recall bias to the data collected from the investigators. The retrospective assessment is also a limitation as requisite data may be missing. These limitations will be addressed with triangulation of data from other sources such as project reports and administrative records and extensive sensitivity testing in the economic assessment. The retrospective application of FAIT is not ideal but will validate the use of the Framework for completed research studies. Reliance on interviews with the CIs, as opposed to also including end-users, is another possible limitation. However, research conducted by Donovan et al. [20] has shown that, rather than overestimate impact, CIs may underreport the impact of their research, as has been noted in previous studies [21, 22]. Again, triangulation of data will be used to mitigate this limitation. One further challenge is expected in undertaking the cost benefit analysis to value the return on the research investment. While the available data to measure the total cost of the investment are expected to be robust, the data required to populate the domains of benefit are limited. This analysis is further constricted by the absence of a clear counterfactual. The evaluation of some of the interventions comprising Good for Kids lacked controlled designs, hampering attribution of effect in terms of any observed changes in behaviour or weight. As outlined above, there are some data sources, such as data collections from other comparable local health districts or state database collections, which may be helpful in forming an accurate counterfactual. However, in the absence of formal controls, attribution of any benefit will be extensively varied in sensitivity analysis.

It is expected that this study will bring transparency to the pathway between research and/or service delivery to translation into meaningful impact. A better understanding of this path will add to the evidence base on effective research translation activities.
